# Deconstructing progressive inflammatory fibrosis in recessive dystrophic epidermolysis bullosa

**DOI:** 10.15252/emmm.202114864

**Published:** 2021-09-13

**Authors:** Christen L Ebens

**Affiliations:** ^1^ Pediatric Blood and Marrow Transplantation & Cellular Therapies University of Minnesota Minneapolis MN USA

**Keywords:** Genetics, Gene Therapy & Genetic Disease, Immunology, Skin

## Abstract

Recessive dystrophic epidermolysis bullosa (RDEB) is an inherited blistering skin disease, resulting from biallelic mutations in *COL7A1,* the gene encoding type VII collagen (C7). At mucocutaneous barriers, tissue integrity relies upon linked extracellular matrix (ECM) proteins forming a physiologic suture, connecting basal epidermal keratinocytes to the underlying dermis. C7 secreted from epidermal keratinocytes and dermal fibroblasts homotrimerizes in the upper dermis to form anchoring fibrils, a critical component of this suture. Clinical manifestations of RDEB are apparent at birth and include exquisite skin fragility, pain and itch, high metabolic demand, and complications downstream of systemic inflammation. Dermal fibrosis is a critical complication of RDEB. Repeated cycles of mechanical injury and healing trigger characteristic fibrotic changes. In addition to functional limitations from joint strictures and pseudosyndactyly formation, dermal fibrosis in RDEB is a nidus for and potential driver of aggressive squamous cell carcinoma (SCC), the leading cause of death in RDEB. A greater understanding of fibrosis in RDEB promises to inform impactful, life‐prolonging clinical trials in this patient population with no proven systemic therapy or cure.

## Graphical abstract

C. Ebens discusses the study by Bernasconi *et al* (in this issue of *EMBO Mol Med*) that reveals that increased pro‐inflammatory immunity associates with fibrosis evolution in recessive dystrophic epidermolysis bullosa (RDEB) and proposes a therapeutic intervention with Ang‐(1‐7).

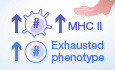

Therapeutic developments in RDEB have largely centered on restoring C7 at the gene or protein level, either via local wound targeting treatments or via systemic therapies to address both external and internal disease manifestations. Local wound therapies under investigation include agents targeting specific *COL7A1* mutations (premature termination codon readthrough agents), topical application of recombinant C7, use of gene‐corrected autologous skin grafts, epidermal allografts following successful allogeneic hematopoietic cell transplantation, intradermal injection of mesenchymal stromal cells, and intradermal injection of gene‐corrected autologous fibroblasts. However, impact of such interventions is limited to the skin, limited in body surface area covered, and limited in duration of benefit. Studies of systemic therapies to restore C7 are fewer in number to date. Until systemic sustained or lifelong therapeutic restoration of C7 is realized, patients with RDEB would benefit from alternative therapies to reduce disease burden and prolong life.

RDEB has been recognized as a disease of systemic inflammation, most apparent in persistently inflamed skin of affected patients, with marked erythema, acute, and chronic wounds at blister sites. Mucocutaneous wound repair is a complex process of cellular recognition of damage and response to restore tissue homeostasis. This process occurs by progression through phases of hemostasis, inflammation, proliferation, and remodeling. Impairment in any or multiple of these wound‐healing phases may result in lack of healing (a chronic wound) or healing with fibrosis. Similar to the above experimental therapies, allogeneic hematopoietic cell transplantation (alloHCT) for RDEB began as a means to provide systemic C7 based on promising pre‐clinical murine investigations. However, clinical response to alloHCT did not correlate with changes in cutaneous basement membrane C7. Interestingly, a population of donor‐derived non‐hematopoietic cells, presumed bone marrow‐derived mesenchymal stromal cells (MSCs), were identified in the dermis (Tolar *et al*, 2011) raising question of an additional benefit of immunomodulation following alloHCT. MSCs are pluripotent cells with long‐term self‐renewal capacity allowing them to play important roles in tissue repair and homeostasis. They home to sites of injury/inflammation and modulate the microenvironment attracting reparative cells to wounded tissue and aiding in appropriate transition from the inflammatory phase of wound healing to proliferative and remodeling phases. MSCs have been shown to inhibit type I inflammatory responses by secretion of interleukin (IL)‐1 receptor antagonist (Harrell *et al*, 2020) and to shift tissue macrophage differentiation toward anti‐inflammatory M2 phenotype (Vander Beken *et al*, 2019). In the context of RDEB, MSC therapies have been investigated as stand‐alone treatments as well as a supplement to alloHCT (Ebens *et al*, 2019).

The clinical benefit demonstrated by immunomodulatory MSCs in treatment of RDEB motivates further dissection of critical cell populations and pathways involved in acute and chronic cutaneous inflammation and ultimately fibrosis development. While circulating plasma levels of cytokines and immune cells is readily obtainable for assessment, tissue evaluations provide the most direct, valuable data. Transcriptomic analysis comparing RDEB to healthy skin has demonstrated increased expression of genes involved in immune system activation (Breitenbach *et al*, 2015). Examinations of wound dressings (Fuentes *et al*, 2020) and blister fluid (Alexeev *et al*, 2017) highlight a plethora neutrophils and lymphocytes recruited by chemokines and chemokine receptor ligands. While several proinflammatory cytokines, chemokines, and cell types have been associated with phenotypic severity in RDEB, few interventional pre‐clinical studies describe successful interruption chronic inflammation with associated decrease in fibrosis.

In 2015, the Nystrom laboratory at the University of Freiburg in Germany elegantly demonstrated inhibition of TGF‐β to reduce fibrosis of chronically injured forepaws in a murine model of RDEB (*COL7A1‐*hypomorphic mice; Nystrom *et al*, 2015). Mass spectrometry‐based tissue proteomics of less affected mouse back skin highlighted baseline elevation of inflammatory markers TNF‐α and IL‐6 in RDEB, reduced upon TGF‐β inhibition. Alternative targeting of TGF‐β with over‐expression of decorin (an endogenous TGF‐β inhibitor) demonstrated similar reduction in fibrosis of *COL7A1‐*hypomorphic mice (Cianfarani *et al*, 2019). Expanding on these earlier findings, Nystrom’s group deployed multiple methods of analysis to unveil provocative age‐ and site‐dependent differences in fibrosis in this same murine model of RDEB, association between inflammation and fibrosis progression, and promising reductions in fibrosis with the use of Ang‐(1‐7) (Bernasconi *et al*, 2021).

The *COL7A1‐*hypomorphic mouse (Fritsch *et al*, 2008) demonstrates progressive fibrosis and early mortality paralleling the clinical course of severe, generalized RDEB in humans. Bernasconi *et al* (2021) identified three time‐points of fibrosis for comparative analysis of the RDEB mice, newborn (early injury), 4 weeks (mid‐stage fibrosis), and 10 weeks (advanced fibrosis) of age. At each time‐point, high‐friction forepaws were compared with low‐friction back skin, RDEB to wild‐type (WT). Mass spectrometry tissue proteomics revealed RDEB forepaws to have increase over time in inflammatory pathway and fibroblast activation. Interestingly, this progressive fibrosis did not correlate with absolute increases in type I collagen but rather ECM remodeling (visually confirmed with picrosirius red staining). In addition to progressive fibrosis, flow cytometric analysis of immune cells in the RDEB skin revealed increase in neutrophils (over time) and CD38^+^ inflammatory macrophages (compared to WT), both cell populations phenotypically activated in forepaws, with high major histocompatibility complex (MHC) II expression. In RDEB mice, adaptive immune changes over time included increased CD4 T lymphocytes. Gene expression analysis of these T‐cell populations shifted from a program of activation (increased *Pdcd1* and *CD27*) at mid‐fibrosis to that of exhaustion (increased *Eomes* and *Pdcd1*) at advanced fibrosis (Fig 1A). These findings of early macrophage infiltrate and increase in T cells over time were replicated in human RDEB skin from different stages of fibrosis/disease.

Recognizing the stimulation of fibrosis by dysregulated inflammation, Bernasconi *et al* (2021) next devised an intervention to target both inflammatory cells and fibroblasts with a naturally occurring anti‐inflammatory heptapeptide, Ang‐(1‐7). Ang‐(1‐7) is a member of the renin‐angiotensin system (RAS), able to activate interconnected fibrosis‐limiting axes of RAS and the kinin‐kallikrein system (KKS). Following dose‐finding studies, *COL7A1‐*hypomorphic mice were treated systemically with one of 2 doses of Ang‐(1‐7) daily for 7 weeks, starting at the clinically relevant time‐point of visible paw deformities (˜5 weeks of age). Remarkably, Ang‐(1‐7) halted fibrosis progression and prolonged survival, with greatest benefit with Ang‐(1‐7) provided at low dose. While no reduction in inflammatory cells was achieved, MHC II expression was decreased on innate immune cells, consistent with a less activated state. Western blots of forepaw tissue lysates demonstrated low dose Ang‐(1‐7) to reduce TGF‐β signaling, transitional ECM proteins and proteins associated with inflammatory potential. Visual assessment of treatment impact with picrosirius red staining found Ang‐(1‐7) associated with less remodeling of structural fibrils. Systemic effects were confirmed with decreases in fibrosis‐associated proteins in major organs affected by fibrosis in RDEB, including the eye, tongue, and esophagus (Fig 1B). Parallel protein analyses of human RDEB fibroblasts and monocytic immune cells demonstrated consistent findings *in vitro*.

**Figure 1:**
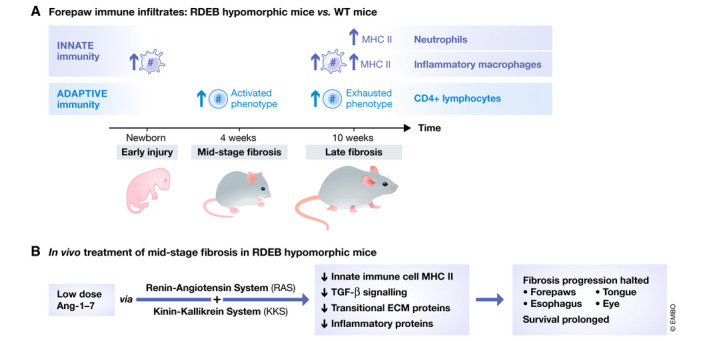
Analysis of time‐dependent immune infiltrate and fibrosis in high‐friction RDEB murine forepaws and impact of intervention with Ang‐(1‐7) (A) Progressive fibrosis correlates with increase in innate immune infiltrate major histocompatibility complex (MHC) II expression and transition from an activated to exhausted phenotype of local adaptive immune cells. (B) Intervention with Ang‐(1‐7) upon onset of forepaw fibrosis engages fibrosis‐limiting axes of the renin‐angiotensin and kinin‐kallikrein systems. In addition to forepaw and systemic decreases in RDEB target organ fibrosis, forepaw inflammatory processes are quelled.

The Nystrom group’s latest work in the pathophysiology of dermal fibrosis in RDEB provides a comprehensive longitudinal view of the interplay between type I immune activation, fibroblast activation, and ECM remodeling, including clinically relevant differences at sites of high vs. low friction. Murine findings were consistent in human samples, further supporting the validity of the *COLA1‐*hypomorphic mouse as a model for human RDEB. Bernasconi *et al* (2021) provide additional robust pre‐clinical data for the use of low dose Ang‐(1‐7) in halting progression of this inflammatory fibrosis. If successfully translated to human patients with RDEB, systemic reduction in progressive fibrosis may allow avoidance of esophageal failure, functional limitations in hand use and mobility, and ultimately may reduce or delay development of life‐threatening SCC. Further, effective reductions in rates of dermal fibrosis may provide a more hospitable niche for future cellular therapies to restore C7 and permit positive clinical impact of the latter at advanced age. While this line of investigation supports strong pre‐clinical data for trials of Ang‐(1‐7) in human RDEB, the potential disease‐modifying benefit of immunomodulatory approaches is also supported.
